# Influence of High Energy Diet and Polygenic Predisposition for Obesity on Postpartum Health in Rat Dams

**DOI:** 10.3389/fphys.2021.772707

**Published:** 2022-02-10

**Authors:** Andrea S. Leuthardt, Julia Bayer, Josep M. Monné Rodríguez, Christina N. Boyle

**Affiliations:** ^1^Institute of Veterinary Physiology, Vetsuisse Faculty, University of Zurich, Zurich, Switzerland; ^2^Laboratory for Animal Model Pathology (LAMP), Institute of Veterinary Pathology, Vetsuisse Faculty, University of Zurich, Zurich, Switzerland

**Keywords:** high energy diet, sucrose preference test, liver steatosis, maternal obesity, leptin resistance, maternal behavior

## Abstract

It is estimated that 30% of pregnant women worldwide are overweight or obese, leading to adverse health effects for both mother and child. Women with obesity during pregnancy are at higher risk for developing both metabolic and mental disorders, such as diabetes and depression. Numerous studies have used rodent models of maternal obesity to understand its consequences on the offspring, yet characterization of changes in the dams is rare, and most rodent models rely solely on a high fat diet to induce maternal obesity, without regarding genetic propensity for obesity. Here we present the influence of both peripartum high energy diet (HE) and obesity-proneness on maternal health using selectively bred diet-resistant (DR) and diet-induced obese (DIO) rat dams. Outbred Sprague-Dawley rats were challenged with HE diet prior to mating and bred according to their propensity to gain weight. The original outbred breeding dams (F0) were maintained on low-fat chow during pregnancy and lactation. By comparison, the F1 dams consuming HE diet during pregnancy and lactation displayed higher gestational body weight gain (*P* < 0.01), and HE diet caused increased meal size and reduced meal frequency (*P* < 0.001). Sensitivity to the hormone amylin was preserved during pregnancy, regardless of diet. After several rounds of selective breeding, DIO and DR dams from generation F3 were provided chow or HE during pregnancy and lactation and assessed for their postpartum physiology and behaviors. We observed strong diet and phenotype effects on gestational weight gain, with DIO-HE dams gaining 119% more weight than DR-chow (*P* < 0.001). A high-resolution analysis of maternal behaviors did not detect main effects of diet or phenotype, but a subset of DIO dams showed delayed nursing behavior (*P* < 0.05). In generation F6/F7 dams, effects on gestational weight gain persisted (*P* < 0.01), and we observed a main effect of phenotype during a sucrose preference test (*P* < 0.05), with DIO-chow dams showing lower sucrose preference than DR controls (*P* < 0.05). Both DIO and DR dams consuming HE diet had hepatic steatosis (*P* < 0.001) and exhibited reduced leptin sensitivity in the arcuate nucleus (*P* < 0.001). These data demonstrate that both diet and genetic obesity-proneness have consequences on maternal health.

## Introduction

Female reproductive health and metabolic status are strongly interconnected. Sufficient energy stores are permissive of ovulation, while pregnancy and lactation represent two metabolically dynamic periods of a female’s life when both the levels of and receptivity to gonadal, placental and metabolic hormones are changing rapidly. Paralleling the global rise in obesity, the prevalence of obesity in women of reproductive age continues to climb. It is estimated that up to 30% of pregnant women worldwide are obese (Fisher et al., 2013; Chen et al., 2018; Singh and DiBari, 2019), which increases the risk of short- and long-term adverse health outcome for both the mother and child (Catalano and Shankar, 2017). Despite this, we still lack a basic understanding of how obesity during pregnancy and lactation can influence the maternal metabolic and behavioral adaptions necessary to bring about new life (Brunton and Russell, 2008).

Proposed by Barker in the late 20th century (The International Society for Developmental Origins of Health and Disease, 2003; Barker, 2007), the developmental origins of health and disease hypothesis (DOHaD) suggests that the maternal environment impacts the long-term health and wellbeing of the offspring. Consistent with this hypothesis, maternal obesity is a significant predictor for childhood obesity, and increases the risk for metabolic syndrome and cardiovascular disease in the offspring later in life (Strauss and Knight, 1999; Catalano et al., 2009; Shrestha et al., 2021). A majority of research employing rodent models of maternal obesity has focused on the intergenerational consequences: a genetic predisposition for obesity, environmental factors, like diet, and their interaction contribute to this intergenerational risk of metabolic disorders (Huls et al., 2021). And while it is documented that women who are overweight or obese during pregnancy are at a higher risk for developing both metabolic and mental disorders, including increased insulin resistance in early pregnancy, gestational diabetes mellitus, hypertension, pre-eclampsia and postpartum depression (Molyneaux et al., 2014; Marchi et al., 2015; Catalano and Shankar, 2017; Kumpulainen et al., 2018), rodent models of maternal obesity have been used less frequently to investigate the precise nature of the biological link between obesity-related factors and the increased risk for these maternal diseases.

A handful of studies have investigated how consumption of a high fat diet (HFD) during pregnancy and postpartum can influence maternal behavioral and physiological parameters in mice and rats. When reported, these studies unequivocally show that consuming HFD during pregnancy leads to increased gestational weight gain (Wahlig et al., 2012; Bolton et al., 2017; Qiao et al., 2019; Moazzam et al., 2021). While several demonstrated that HFD influenced metabolic and behavioral adaptations in the early postpartum period, these findings are not always consistent with one another (Purcell et al., 2011; Bellisario et al., 2015; Bolton et al., 2017; Moazzam et al., 2021), and very few have investigated the influence of diet over the full length of the postpartum period or in the post-weaning phase (Qiao et al., 2019). What is also notable, is that each of these models of maternal obesity utilized HFD to promote increased fat mass prior to, during, or after pregnancy, but almost none considered an inherited, polygenic predisposition for obesity. Thus the question remains unanswered: does the metabolic state of obesity directly cause the elevated risk for these maternal disorders, or is rather the lifestyle or genetic factors, which contribute to obesity, that predispose these mothers to adverse health outcomes?

Decades of work in rodent dams has revealed numerous metabolic and behavioral adaptations essential for successful gestation of and caring for offspring, as well as the neural networks orchestrating them. Two exemplar adaptive systems involve the hormones leptin and oxytocin. The induction of gestational leptin resistance encourages positive energy balance during pregnancy, which is essential to support the forthcoming energy demands of late-pregnancy fetal growth and lactation (Seeber et al., 2002; Ladyman and Grattan, 2004; Grattan et al., 2007). Oxytocin-producing neurons, which are found in the supraoptic and paraventricular nuclei of the hypothalamus, undergo morphological and functional changes during pregnancy in order to promote parturition and lactation (Augustine et al., 2018). Oxytocin is important for the initiation of maternal behavior (Pedersen et al., 1982), and also acts as a controller of energy balance (Lawson, 2017). Yet, despite recognizing the interconnectedness of metabolic status and reproductive health, there are little data describing whether these, and other hormonal, adaptations are modified in the presence of maternal obesity.

In 2018, our laboratory set out to establish new colonies of lean diet-resistant (DR) and diet-induced obese (DIO) rats, selectively bred based on their propensity to gain weight when maintained on a palatable, high-energy diet (HE). Following protocols originally described and developed by Levin et al. (1997), the DR and DIO colonies were derived from outbred Sprague-Dawley rats, which express greater epigenetic and genetic variability than inbred strains, and therefore mimic most instances of human obesity, which are polygenic in nature (Bell et al., 2005; Madsen et al., 2010; Nilsson et al., 2012; Giles et al., 2016). Over eight generations of breeding, we had the unique opportunity to assess various physiological and behavioral parameters in the breeding dams, to explore whether consumption of HE diet or a DIO phenotype contribute to metabolic and behavioral aberration in the postpartum period. Our long-term goal is two-fold. First, to broadly characterize a rat model of maternal obesity that incorporates polygenic susceptibility in an effort to better simulate most human cases of obesity, compared to models that rely solely on HFD. Second, to further use this exploratory characterization to generate and test hypotheses on how the metabolic challenge of obesity, during the already metabolically challenging periods of pregnancy and lactation, can influence the maternal body and brain in ways that increase vulnerability for various metabolic and mental diseases in the postpartum period and beyond. Here we present an initial characterization of this polygenic model of maternal obesity.

## Materials and Methods

### Selective Breeding of Diet-Resistant and Diet-Induced Obese Rats

**FIGURE 1:**
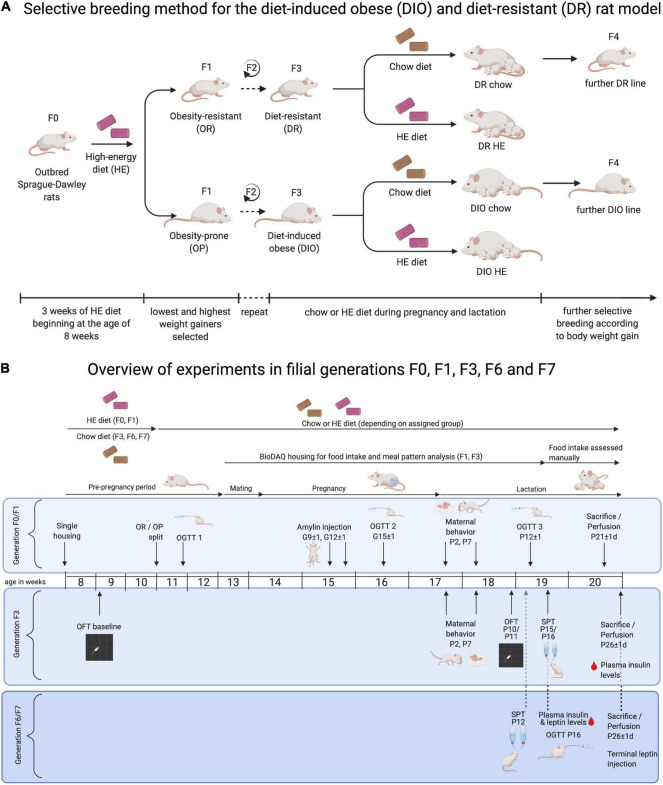
Overview of protocol for selective-breeding of DIO and DR rats and the timelines of experimental procedures in pregnant and lactating rat dams. **(A)** Outbred Sprague-Dawley rats (F0) were used to generate selectively bred colonies of diet- resistant (DR) and diet-induced obese (DIO) rats, according to protocols developed by Levin and colleagues. Following a 3-week challenge on sweetened high-energy diet (HE), rats were classified as obesity-resistant (OR) or obesity-prone (OP) based on their weight gain. OR and OP male and female rats were mated, and the process was repeated in F1 and F2 offspring. Rats were designated as DR and DIO from F3 forward. For breeding and experimental purposes, F3, F6, and F7 DR and DIO dams were maintained on chow or HE from the time of mating and until the time of pup-weaning. Offspring from chow-fed dams were used for colony maintenance. **(B)** Three experiments were carried out in dams from F0/F1, F3, and F6/F7 generations, respectively. Maternal body weight and food intake were collected at least weekly over the course of the studies. Experimental procedures were performed on the gestational (G) and postpartum (P) days indicated, including: oral glucose tolerance test (OGTT); amylin sensitivity test; pup retrieval and maternal behavior test; open field test (OFT); and sucrose preference test (SPT). Between P21 and P26, rat dams were perfused, and blood, brains, and livers were harvested for further analysis. Diagrams were created with a paid-subscription of BioRender.com.

Newly established selectively bred DR and DIO lines were initially derived from 50 male and 50 female outbred Sprague Dawley rats (RjHan:SD, Janvier Labs; Le Genest-St-Isle; France), using the protocols of Levin et al. (1997). At 8 weeks old, all rats were single-housed and provided *ad libitum* access to high energy diet (HE, Open Source Diets D12266B; 4.41 kcal/g, see Table 1 for macronutrient composition) for 3 weeks. The term “HE” will be used to specifically refer to this diet, while “HFD” will be used to refer more generally to high fat diets (typically between 45 and 60% from fat) used in previously published experiments. Body weight gain of each rat during the HE-challenge was ranked. For each sex, the 15 lowest weight-gaining rats were identified as obesity-resistant, and the 15 highest weight-gaining as obesity-prone. After at least 1 week maintenance on standard rodent chow (KLIBA 3436; 3.14 kcal/g, see Table 1 for macronutrient composition), 12 obesity-resistant (OR) and 13 obesity-prone (OP) breeding pairs were created and designated as F0. The same HE-challenge and OR/OP selection procedure was carried out for the F1 and F2 generations. Beginning with F3, the breeding pairs were semi-randomly selected (taking average-weight pups free of abnormalities at weaning) without prior HE exposure, and the mating of rats who shared first or second-degree relatives was avoided. Based on nomenclature of Levin et al. (1997), rats from F3 and forward were designated as DR and DIO. The selective-breeding procedure is depicted in Figure 1A.

**TABLE 1:** Macronutrient composition of experimental rodent diets.

	Chow diet, 3436 Provimi Kliba	High energy diet, Open Source Diets D12266B
Caloric density	3.14 kcal/g	4.41 kcal/g
% kcal from fat	12.3	32.5
% g from fat	4.5	15.6
% kcal from protein	22.3	16
% g from protein	18.5	17.6
% kcal from carbohydrate	65.4	43.3*
% g from carbohydrate	54	47.6*
		*50.8% of carbohydrate from sucrose

For some experimental groups, female dams were maintained on HE from the time of mating until the time of pup weaning. With the exception of pups derived from F1 dams, only pups derived from chow-fed dams were used to generate subsequent breeding pairs. Date of parturition was designated as postpartum day 1 (P1). In the first study dams and pups were not disturbed on P1, but in later generations dam body weight was measured on P1. On P2, maternal body weight, litter weight, size and sex ratio were recorded. Litters were then culled to 10 pups, consisting of 5 male and 5 female pups, when possible. If a dam gave birth to less than 10 pups, we cross-fostered pups from the same experimental group (matching diet and phenotype). Pups were weaned from the dam between P21 and P26.

In an effort to promote the 3R principles, the number of breeding pairs created for each generation was calculated based on the existing need for DR and DIO rats for other experiments in our laboratory. As pups born to dams consuming HE during pregnancy and lactation have known developmental disruptions (Bouret et al., 2008), and are therefore less suitable for breeding colony maintenance, we limited the number of HE-fed dams in our study in an effort to reduce the number of surplus offspring. Reduced pregnancy success rates also contributed to smaller group sizes (See Supplementary Table 1). If, after assigning weanlings to colony breeding or other experiments, there was a surplus, we made every effort to re-home female rats through the Animal Welfare Office at the University of Zurich. Surplus rats that were not re-homed were killed and donated to the Zurich Zoo.

### Experimental Subjects

For the present study, data were collected from rat dams from generations F0, F1, F3, F6, and F7, which were used to investigate the influences of maternal intake of HE diet and the polygenic sensitivity to this diet on maternal health parameters (described below):

#### Experiment One

**FIGURE 2:**
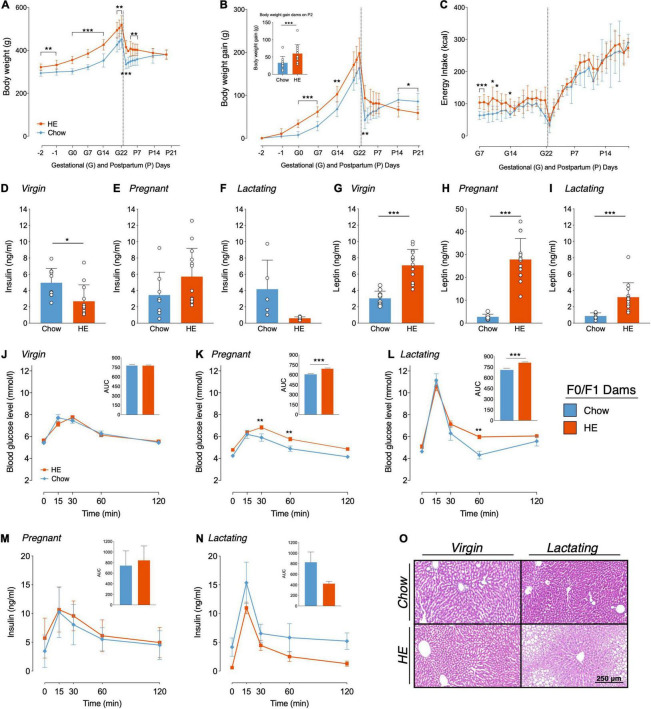
Influence of HE-diet on physiological parameters in obesity-prone and -resistant rat dams in filial generation 0 and 1 (F0/F1) before, during and after pregnancy. Body weight in grams **(A)**, body weight gain in grams **(B)** and energy intake in kilocalories **(C)** of Sprague Dawley dams on chow (blue) or high energy (HE, orange) diet over the course of pregnancy and lactation. Vertical dotted line marks parturition. **(B)** Inset shows body weight gain during pregnancy (difference between body weight on gestational day 1 (G1) and P2 to account for litter and placenta weight). **(D–I)** Plasma insulin and leptin levels (ng/ml) following a 12 h fast in dams on chow and HE diet. **(D,G)** Insulin and leptin levels in virgin dams following a 1 week HE challenge. **(E,H)** Insulin and leptin levels during mid-pregnancy (gestational day 15). **(F,I)** Insulin and leptin levels in mid-lactation (postpartum day 12). **(J–L)** Blood glucose levels (mmol/l) during an oral glucose tolerance test in virgin **(J)**, pregnant **(K)**, and lactating **(L)** dams on chow or HE diet. Inset in each graph **(J–L)** shows the area under the curve relative to zero. Insulin levels (ng/ml) during the OGTT in pregnant **(M)** and lactating **(N)** rats on either chow or HE diet. Insets show area under the curve relative to 0 ng/ml. **(O)** Representative HE-stained liver sections of virgin and lactating dams on either chow or HE diet (Scale Bar: 250 μm). Data are represented as mean ± SD. *P < 0.05, **P < 0.01, ***P < 0.001. *N* = 10 in chow group, *N* = 14 in HE group.

Our initial examination of metabolic and behavioral parameters between OR and OP dams maintained on chow (F0) or HE (F1) yielded few differences between the phenotypes (data not shown). Low pregnancy success rates in F0 further hindered these comparisons (See Supplementary Table 1). However, we observed robust effects of HE-intake during pregnancy and lactation in these early generations. We therefore compared data from all chow-fed dams in F0 (*n* = 10) to those obtained from obesity-prone HE-fed dams in F1 (*n* = 14). Selection of these groups was based on their clear separation of pre-mating fasting plasma leptin concentrations, taken after the initial HE challenge (See data in Figure 2G).

#### Experiment Two

The effects of the selective-breeding became more evident in F3 dams, and comparisons were made between DR-chow (*n* = 10), DR-HE (*n* = 5), DIO-chow (*n* = 9), and DIO-HE (*n* = 5) dams. This experiment focused exclusively on the postpartum period.

#### Experiment Three

Select parameters were again assessed in later generations of the DR and DIO rat dams, specifically from generations F6 [DR-chow (*n* = 8), DR-HE (*n* = 7), DIO-chow (*n* = 8), and DIO-HE (*n* = 6)] and F7 [DR-chow (*n* = 6), DR-HE (*n* = 6), DIO-chow (*n* = 6), and DIO-HE (*n* = 4)]. This experiment focused exclusively on the postpartum period.

An overview of the timelines for each experiment is shown in Figure 1B. All experiments were performed with the approval of the Veterinary Office of the Canton Zurich, Switzerland, and in accordance with the European Union Directive 2010/63/EU on the protection of animals used for scientific purposes.

### Measurement of Body Weight Gain, Food Intake and Meal Patterns

For dams from the generations F0, F1, and F3, dam body weight and litter weight were recorded at least weekly, and daily between gestational day 20 (G20) and P4. Daily energy intake in kcal was measured with the BioDAQ food intake monitoring system. The data were monitored with BioDAQ-E2 software (2.3.01 2011.02.10; New Brunswick; United States). For F0 and F1 dams, 24-h energy intake data was analyzed to determine differences in meal patterns between pregnant (on G7 and G20 ± 1) and lactating (P4, P9, and P15 ± 1) rats, either fed with chow diet or HE. To analyze meal pattern information, the data was grouped into separate meals consisting of clustered feeding bouts. A meal was defined by a minimum inter-meal interval (IMI) of 900 s and a minimum meal size of 0.23 g (Duffy et al., 2018). Total energy intake (in kcal), average meal size (in kcal) and average meal number were calculated. Following the same procedures, meal patterns of F3 dams were analyzed on G20, P4, P9, and P12. For dams from generations F6 and F7, weekly body weight was measured from date of mating until sacrifice when the pups were weaned.

### Amylin Sensitivity Test

In the second week of pregnancy (G11 ± 2), F0 and F1 dams were tested for amylin sensitivity. Following a 4-h fast, a single dose of amylin (20 μg/kg; amylin trifluoracetate salt; Bachem, CH Catalog# 403020; Lot# 1064269) or vehicle (saline) was administered subcutaneously just before the onset of the rats’ active dark phase. Our earlier work showed that this dose of amylin reduces food intake and activates central brain structures mediating amylin’s effects (Boyle et al., 2011; Honegger et al., 2021). Rats were tested over 2 trials in a randomized crossover manner, with a 48-h washout between trials. Food intake was measured 30 min, 1, 2, 4, and 24 h after injection using the BioDAQ food intake monitoring system.

### Oral Glucose Tolerance Test

In F0/F1 dams, oral glucose tolerance tests (OGTTs) were performed in virgin, pregnant (G15 ± 2) and lactating (P12 ± 1) rats. Prior to the first OGTT, rats were trained to drink a 30% glucose solution from a syringe. Rats were fasted for 12 h in the light phase prior to the test. A baseline sample was obtained from tail blood, for the measurement of glucose and subsequent analysis for leptin and insulin. Rats were then given the glucose load orally (2.5 g/kg; 8.3 mL/kg consumed in 5 min), and tail blood glucose levels were measured at 15, 30 min, 1, and 2 h using a glucometer (Contour XT; Ascensia Diabetes Care; CH). At each time point, a 100-μl blood sample was collected for insulin determination. Blood samples were collected in Na-EDTA-coated tubes (Microvette; 100 μl; Sarstedt) containing a general protease inhibitor cocktail (P2714; Sigma; 30 μl of 1:10 diluted stock solution/1 ml blood). Samples were kept on ice until spun for 10 min at 13’200 rpm (4°C), and the plasma was immediately aliquoted and stored at −80°C until assayed for insulin and leptin plasma levels. Total area under the curve (AUC) was calculated from 0 for glucose and insulin data using the trapezoidal rule.

Oral glucose tolerance test was also performed in F6/F7 dams. We attempted the first OGTT in F6 dams on G15, however we observed that the pregnant DIO dams, regardless of diet, refused to orally consume the 30% glucose solution. While DR dams consumed an average of 87% of their calculated dose, DIO dams consumed 47%. Glucose (2.5 g/kg) was therefore administered via gavage for the OGTT performed on P18 ± 1 for F6 and F7 dams.

### Measurement of Leptin and Insulin

Leptin and insulin were measured in duplicate in plasma using the MSD^®^ U-Plex Platform Multiplex Assay (U-PLEX Metabolic 2-Plex Combo 1 (Rat), Catalog# K15312K-2, Lot# 348956); plates were analyzed using the MESO QuickPlex SQ 120 imager. Hormones were measured in the OGTT baseline samples for F0, F1, F6, and F7 dams (after a 12-h fast), and in terminal samples for F3 (after a 2-h fast; treated with leptin).

### Behavioral Parameters

#### Maternal Behavior Tests

To assess maternal motivation, a pup retrieval test followed by 60-min of home cage observation was performed in the F0/F1 and F3 dams on P2 and P7. Maternal behavior tests were conducted in the first 4 h after dark onset. Before each test, location of the pups and nest was recorded, and then pups were placed in a holding cage under a warming light for 10 min. The test was initiated when 5 pups were placed in each of the two corners of the home cage opposite of the nest.

For tests with F0/F1 dams, maternal behaviors were manually recorded in real-time with a stop-watch. The latency to retrieve the first pup and all pups, and the latency to assume a nursing position was recorded. Subsequently, the dam’s behavior was recorded every minute for 60 min. Each behavior was assigned to one or maximum two of the following categories: retrieving, nursing (further defined as hover, high crouch, or low crouch; See (Stern and Johnson, 1990) for reference), pup-grooming, self-grooming, eating, drinking, exploring, or quiet (out of the nest). Using this manual scoring, the resolution of these data was at the level of minutes.

The primary aim for the F3 generation was to perform a higher resolution analysis of maternal behaviors than was obtained during F0/F1. The general timeline matched the F0/F1 maternal behavior test, but to achieve a finer detail, the 60-min test was recorded using a RaspPi Camera. Up to 6 dams were tested in a single session, and the behaviors of the dams were blindly scored using the free and open-source software Behavioral Observation Research Interactive Software (BORIS) (Friard and Gamba, 2016). The following behaviors were analyzed: latency to retrieve first pup, latency to retrieve all pups, nursing duration, nursing positions (hover, low crouch and high crouch), nest building, pup grooming, self-grooming, eating, drinking, exploring and quiet (out of the nest). These behaviors were sub-categorized as pup-directed or self-directed behaviors. Pup retrieval, pup grooming, nursing and nursing position accounted for the pup-directed behavior, whereas self-directed behavior included self-grooming, eating, drinking, exploring and quiet. Additional details of the analysis can be found in the Supplementary Methods. By scoring the videos using BORIS, duration of each behavior during the test could be evaluated at the level of seconds.

#### Open Field Test

In F3 dams, open field tests (OFT) were conducted in each female rat prior to mating or access to HE diet (baseline, at approximately 9 weeks of age), and again on postpartum day 10 or 11. Tests were performed during the light phase, between 8 and 11 am, in a testing room separate from the animal housing room. The OFTs were conducted in an open metal frame (80 cm × 79 cm × 40 cm). The floor of the frame was covered with a transparent plastic floor to facilitate cleaning, under which black plastic was placed to increase contrast of white rat in the frame. Dams were always placed into the same corner of the square and immediately left alone in the room. The behavior and movement of the animal was recorded for 15 min with a video camera (RaspPi Camera). After the test the rat was brought back to her home cage, the floor and walls of the Open Field were cleaned with Virkon™ S and water, and dried.

Videos were analyzed using ezTrack (Pennington et al., 2019), a free and open source program using Jupyter notebook and interactive Python scripts (Python 3.7.). All required programs used for analysis were from the Denise Cai Lab ezTrack Github^1^. Data were summarized using binned summary reports. Bins were defined as frames: 0–27000; 0–9000; 9001–18000; 18001–27000, which represent 0–15 min of the video, 0–5, 5–10, and 10–15 min. For each bin the following parameters were analyzed (all data in centimeters unless defined otherwise): Total distance, total distance per bin, total periphery zone distance, total center zone distance, center zone distance per bin, percentage of total time spent in center, total number of center entries, total number of center zone entries per bin.

#### Sucrose Preference Test

Beginning at postpartum day 12, 4 consecutive days of sucrose preference testing (SPT) were conducted with rat dams maintained in their home cages. The standard water bottle (500 ml) was replaced with two smaller water bottles (250 ml each); one containing 1% sucrose solution (Sucrose ACS reagent, Sigma-Aldrich) and the other containing water. Each bottle was weighed and refilled every day. The left-right placement of the bottles was changed each day to distinguish a side preference from the actual sucrose preference. For each day of testing, any preference for or avoidance of the sucrose solution was calculated as percentage of the total volume consumed: [100× volume of sucrose solution consumed/total volume consumed]. The intake of sucrose and water on the last 2 days of testing were averaged. Data from rats demonstrating a consistent side-preference (drinking 75% or more from the same side) were not included in the analysis.

### Immunohistological Analysis of Postpartum Brain Tissue

#### Perfusion and Tissue Preparation

Rat dams were sacrificed between postpartum days 21 and 26. On the morning of perfusion, pups were separated from dams at least 1 h prior to any treatment, at which time food was removed from the cage. To test for central leptin sensitivity, dams from F3 and F7 were treated with leptin prior to perfusion. Within the experimental groups, dams were randomly assigned to receive PBS vehicle or leptin (i.p., Murine leptin; G2817 Lot: 071776; PeproTech; United Kingdom; Diluted in 0.01 M PBS, pH 8.0). Dams from F3 were treated with 2 mg/kg, but since this failed to induce pSTAT3 in the hypothalamus, F7 dams were subsequently treated with 5 mg/kg. Forty-five minutes after leptin or vehicle injection, rats were injected with an i.p. injection of 300–450 mg/kg Pentobarbital (Esconarkon ad us. Vet.; 300 mg/ml; Streuli Pharma AG; Uznach CH). When deeply anesthetized, the abdominal and thoracic cavities were opened, a blood sample was taken from the right ventricle with an 18-G needle, transferred to a Na-EDTA coated tube, and kept on ice before centrifuged for 10 min at 13’200 rpm. Plasma was aliquoted and directly frozen on dry ice. Following blood collection, samples were collected from the right median lobe of the liver and periovarian adipose tissue; one portion of each were snap-frozen in liquid nitrogen, and one portion stored in 4% paraformaldehyde (PFA) for histological analysis. The rat was then flushed by hydrostatic forces with 0.1 M ice cold phosphate buffer (PB) for 1.5 min followed by a 2.5-min fixation with ice-cold 2% PFA. The brain was extracted and put into 2% PFA on ice. In some cases, liver samples were collected after perfusion, and the resulting in dilated liver sinusoids (See Supplementary Material for discussion). Brains were transferred to 20% sucrose solution in 0.1 M PB for 24 h in a 4°C cold room. Brains were blocked into fore- and hindbrain, frozen in hexane on dry ice for 4 min, and stored at −80°C until sectioning.

#### Immunohistochemistry in Brain

Frozen forebrains of DIO/DR generation F7 were sectioned in four series of 25 μm thickness on a cryostat (Leica CM3050 S; Biosystems; DE). Sections were mounted directly onto Superfrost^®^ glass slides (Superfrost^®^ Plus; Thermo Scientific; DE), beginning at +0.2 mm from bregma through the hypothalamus until −3.25 mm from bregma, according to the Swanson Brain Maps Atlas (Swanson, 2004). Between −0.83 and −1.08 from bregma a total of 4 sections (200 μm in total) were discarded. All other sections were collected. Slides were stored at −20°C in cryoprotectant solution [20% glycerol; 30% ethylene glycol; 50% phosphate buffer (0.1 M PB)] until staining.

### Oxytocin and pSTAT3 Immunostaining

Two series of the brain sections were separately analyzed for oxytocin and pSTAT3 expression using immunofluorescence protocol optimized for the visualization of pSTAT3 [see (Levin et al., 2004) for details]. In brief, slides were blocked in 4% NDS, 0.4% Triton and 1% BSA in KPBS for 20 min and then incubated in rabbit-α-pSTAT3 (1:500; 9145 from Cell Signaling Technology) or mouse-α-Oxytocin (1:1000; Cat # MAB5296; Lot # 3061232; Chemicon; Temecula, CA, United States) in 1% NDS, 0.4% Triton and 1% BSA in KPBS for two overnights at 4°C. After primary incubation slides were rinsed in KPBS, followed by a secondary incubation step in donkey-α-rabbit–Cy3 or donkey-α-mouse–Cy3 (both 1:100; Jackson ImmunoResearch, Laboratories) in 1% NDS and 0.3% Triton in KPBS for 2 h at room temperature protected from light. Slides were rinsed again in KPBS, counterstained in DAPI for 4 min and washed in KPBS. After 30 min air drying slides were cover-slipped with Vectashield^®^ (Vectashield^®^ Hardset™; Antifade Mounting Medium for Fluorescence; Ref # H-1400; Lot # ZH0114). Slides were stored at 4°C in the dark until further analysis.

### Fluorescence Microscopy and Image Analysis

Fluorescence images were taken at 10× magnification for oxytocin neurons and at 20× magnification for pSTAT3 neurons with a Zeiss Axio Camera HRm (AxioCam HRm, Carl Zeiss Microimaging GmbH, Göttingen, Germany). Sections were excited at 550 nm to visualize pSTAT3- or oxytocin-positive neurons depending on the slide series.

Images were analyzed using Image J software [Version 2.0.0-rc-69/1.52p; open source image processing software; (Schindelin et al., 2012)] together with short macro programs customized for quantification of pSTAT3- or oxytocin-positive neurons. For analysis, 8-bit grayscale pictures were used. Threshold for pSTAT3 neuron count was set at 38–255 and the size of counted cells was defined as 30 square pixels up to infinity. For the evaluation of oxytocin cells, first the function “Gaussian Blur…” with a sigma value = 2 was applied. Threshold was then set at 40–255, size was set at 40 square pixels up to infinity and the application “Watershed” was used to divide overlapping cell bodies. For oxytocin, the macro was run on brain sections containing paraventricular nucleus of the hypothalamus [PVH, corresponding to Levels 24 through 27 in Swanson (2004)]. For pSTAT3, brain sections containing arcuate nucleus of the hypothalamus [ARH, Levels 28 through 30 in Swanson (2004)] were analyzed.

### Histological Analysis of Postpartum Liver

Liver tissue samples collected from F0/F1, F3, and F7 dams were sent to the Institute of Veterinary Pathology at the University of Zurich for further evaluation. Liver tissue was embedded into a paraffin block, sliced in 3–5 μm thick sections and mounted onto glass slides. Following hematoxylin and eosin-staining (H&E), slides were scanned using a slide scanner (NanoZoomer-XR C12000; Hamamatsu, Hamamatsu City, Japan). Liver sections were semi-quantitatively scored using the non-alcoholic fatty liver disease (NAFLD) Clinical Research Network Scoring System (Kleiner et al., 2005), taking into account that NAFLD in rodents is not associated with the development of megamitochondria and a few other features described in human NAFLD (Liang et al., 2014). Under this steatosis scoring system, a score of 0 represents not present, 1 represents very mild, 2 represents mild, 3 represents moderate, and 4 represents severe steatosis.

### Statistical Analysis

For all statistical analyses Microsoft^®^ Excel (version 16.48) and Prism 9 (version 9.1.0) were used. Depending on the dataset, mixed-effect model analysis, two-way-ANOVA or unpaired parametric two-tailed Student’s *t*-tests were used to evaluate the influence of diet (chow vs. HE), phenotype (DR vs. DIO), and time on each parameter measured. To account for missing values in a repeated measures ANOVA, a mixed-effect model analysis was used. For multiple comparisons (*post hoc* tests), a Tukey’s or Sidak’s multiple comparison was used. To analyze ordinal data (semi-quantitative NAFLD scores), scores were averaged for each group and non-parametric Kruskal-Wallis tests were used, followed by Dunn’s planned multiple comparison test. *P*-values ≤ 0.05 were defined as statistically significant. Data are represented as mean ± SD.

## Results

The process of the establishing selectively bred DR and DIO colonies evolved from generation to generation. The results are therefore presented in three parts, each representing the observations made from dams in the early stage of selective breeding (F0 and F1 dams), mid-stage of selective breeding (F3 dams), and later stages of selective breeding (F6 and F7 dams).

### Experiment One: Influence of High Energy Diet on Rat Dams (F0 and F1) During Pregnancy and Lactation

#### Maternal Body Weight Gain and Food Intake

Compared to dams eating chow, maternal intake of HE resulted in differences in body weight (Figure 2A) and body weight gain (Figure 2B), which were especially pronounced during gestation and the first week of lactation. Main effects of diet (*F*_1_,_22_ = 27.25, *P* < 0.001) and time (*F*_2_._60_,_53_._19_ = 294.0, *P* < 0.001) on body weight were found, with significantly higher body weight in HE-fed rats at all time points excluding P14 and P21 (Figure 2A). Furthermore, a significant interaction was detected (*F*_15_,_307_ = 12.57, *P* < 0.001). Main effects of diet (*F*_1_,_21_ = 6.592, *P* = 0.018) and time (*F*_2_._61_,_53_._76_ = 218.8, *P* < 0.001) on body weight gain were found, with significantly increased body weight gain in HE-fed rats compared to chow-fed rats during early to mid-pregnancy, and on P2, P14, and P21 (Figure 2B). Comparison of body weight gain during pregnancy (the difference between body weight on G1 and on P2 to account for litter and placenta weight; See inset in Figure 2B) revealed that dams fed HE gained significantly more weight than chow-fed dams during pregnancy (*t*_21_._99_ = 2.925, *P* = 0.008). HE also influenced the caloric intake during pregnancy and postpartum: Main effects of diet (*F*_1_,_21_ = 9.32, *P* = 0.006) and time (*F*_7_._86_,_172_._6_ = 130.2, *P* < 0.001) were observed, with individual differences observed in early and mid-pregnancy (Figure 2C).

#### Leptin and Insulin Levels

Insulin and leptin were measured in plasma following a 12-h fast and prior to an OGTT performed before mating (1 week following the HE-challenge), during mid-pregnancy (G15), and mid-lactation (P12). Prior to mating, insulin values were lower in the prospective HE-fed females than those maintained on chow (*t*_20_ = 2.774, *P* = 0.012; Figure 2D), but were not different during pregnancy (Figure 2E) or lactation (Figure 2F). Leptin, however, was significantly elevated in the HE-fed females prior to (*t*_19_._22_ = 6.851, *P* < 0.001; Figure 2G), during (*t*_13_._69_ = 10.14, *P* < 0.001; Figure 2H), and after pregnancy (*t*_14_._03_ = 4.526, *P* < 0.001, Figure 2I).

#### Oral Glucose Tolerance Test

Intake of HE also influenced glucose clearance during an OGTT in pregnant and lactating, but not virgin, rat dams. In virgin rats a main effect of time (*F*_4_,_88_ = 99.3, *P* < 0.001) was found as well as a significant interaction between time and diet (*F*_4_,_88_ = 2.886, *P* = 0.027; Figure 2J). In pregnant rats (Figure 2K), main effects of diet (*F*_1_,_21_ = 24.58, *P* < 0.001) and time (*F*_4_,_84_ = 45.62, *P* < 0.001) were detected, with HE-fed rats exhibiting higher blood glucose levels than chow-fed rats at 30 min (*t*_105_ = 3.351, *P* = 0.006) and 60 min (*t*_105_ = 3.2, *P* = 0.009) after the glucose challenge. During lactation (Figure 2L), main effects of diet (*F*_1_,_21_ = 7.414, *P* = 0.013) and time (*F*_4_,_84_ = 98.29, *P* < 0.001) were observed, as well as a significant interaction (*F*_4_,_84_ = 2.627, *P* = 0.04). Significantly higher blood glucose levels in HE-fed dams compared to chow-fed were found at the 60 min time point (*t*_105_ = 3.389, *P* = 0.005). AUC analysis, relative to zero, demonstrated significantly higher AUCs in pregnant and lactating HE-fed rats compared to pregnant or lactating chow-fed rats, respectively (Pregnancy *t*_21_ = 5.176, *P* < 0.001, Lactation *t*_21_ = 4.3, *P* < 0.001; Figures 2K,L insets).

Insulin levels during the OGTTs performed in pregnant and lactating dams displayed a similar curve pattern as blood glucose levels, with a peak in plasma insulin concentration 15 min after the glucose challenge (Figures 2M,N). In pregnant rats, only a main effect of time (*F*_4_,_76_ = 25.4, *P* < 0.001) was found, whereas in lactating rats main effects of diet (*F*_1_,_11_ = 8.592, *P* = 0.014) and time (*F*_4_,_44_ = 23.34, *P* < 0.001) were detected. However, no significant differences in plasma insulin concentrations were found comparing pregnant or lactating chow-fed rats to pregnant or lactating HE-fed rats. AUC, relative to 0 ng/ml, did not show any significant differences in insulin concentration between chow- and HE-fed rats.

#### Liver Histology

We observed no influence of HE diet following histological microscopic inspection of visceral adipose tissue collected from F0/F1 dams (data not shown). In the livers, however, we observed mild to severe steatosis in 100% of the livers from HE-fed lactating dams (average score was 1.93 ± 0.83), while 100% of chow-fed dams received a score of 0, indicating no hepatic steatosis. What was perhaps most interesting, was the observation that three female rats that were maintained on HE diet for the same duration as the dams, but who did not become pregnant, also all received a score of 0, suggesting that hepatic steatosis resulted from an interaction between HE consumption and the states of pregnancy and lactation. Representative images from virgin or lactating females, fed chow or HE, are shown in Figure 2O.

#### Meal Patterns

**FIGURE 3:**
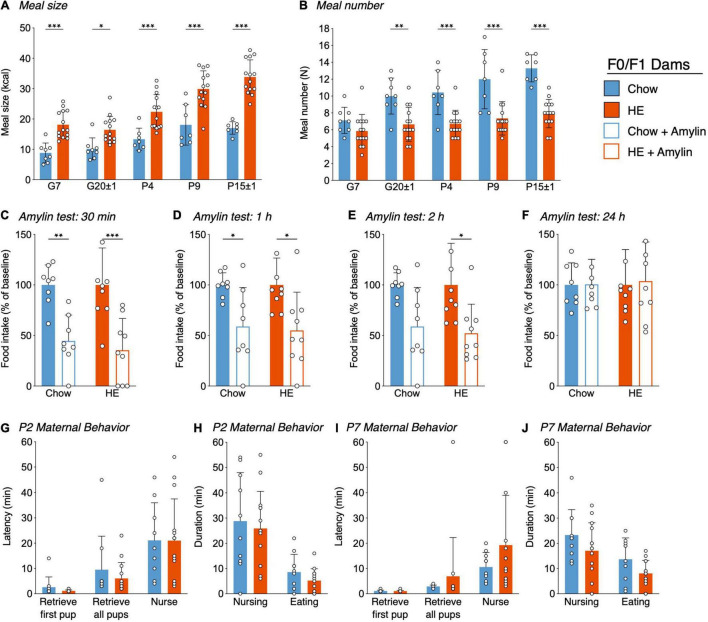
Influence of HE-diet in obesity-prone and -resistant rat dams in filial generation 0 and 1 (F0/F1) on meal pattern, amylin sensitivity, and maternal behavior. Average meal size in kilocalories **(A)** and meal number **(B)** of pregnant and postpartum rat dams on chow or high energy (HE) diet on gestational day 7 (G7), G20 ± 1, postpartum day 4 (P4), P9, and P15 ± 1. **(C–F)** Food intake as percentage of baseline following a single dose subcutaneous amylin (20 μg/kg) or vehicle (saline) injection in rat dams on gestational day 11 ± 2, 30 min **(C)**, 1 h **(D)**, 2 h **(E)**, and 24 h **(F)** after injection. **(G–J)** Pup retrieval test and 60 min maternal behavior home cage observation in dams on either chow or HE diet on P2 and P7. Latency to retrieve first pup, latency to retrieve all pups and latency to start nursing in minutes on P2 **(G)** and P7 **(I)**. Total nursing and eating duration in minutes on P2 **(I)** and P7 **(J)**. Data are represented as mean ± SD. *P < 0.05, **P < 0.01, ***P < 0.001. *N* = 10 in chow group, *N* = 14 in HE group.

Analysis of meal size (Figure 3A) revealed a main effect of diet (*F*_1_,_97_ = 113.4, *P* < 0.001) and time (*F*_4_,_97_ = 26.22, *P* < 0.001) as well as significant interaction (*F*_4_,_97_ = 3.183, *P* = 0.018). Meal size was significantly larger in HE-fed rats than in chow-fed rats for all pregnant and postpartum measurement days: G7 (*t*_97_ = 4.305, *P* < 0.001), G20 ± 1 (*t*_97_ = 2.905, *P* = 0.024), P4 (*t*_97_ = 4.449, *P* < 0.001), P9 (*t*_97_ = 5.696, *P* < 0.001), and P15 ± 1 (*t*_97_ = 7.659, *P* < 0.001). Meal number (Figure 3B) was significantly lower in HE-fed rats compared to chow-fed rats. Main effects of diet (*F*_1,97_ = 77.98, *P* < 0.001) time (*F*_4,97_ = 11.31, *P* < 0.001), and an interaction (*F*_4,97_ = 2.85, *P* = 0.028) were detected. Meal number was significantly decreased in HE-fed rats compared to chow-fed rats on G20 ± 1 (*t*_97_ = 1.40, *P* = 0.002), P4 (*t*_97_ = 3.71, *P* < 0.001), P9 (*t*_97_ = 4.92, *P* < 0.001), and P15 ± 1 (*t*_97_ = 5.67, *P* < 0.001).

#### Amylin Sensitivity Test

Because pregnancy causes reduced sensitivity to hormones regulating food intake, including leptin and cholecystokinin (Ladyman and Grattan, 2004; Ladyman et al., 2011), we assessed if the action of the satiating hormone amylin is preserved in pregnant rat dams maintained on either chow or HE diet (Figures 3C–F). By representing the food intake following amylin-treatment as a percentage of baseline (vehicle food intake = 100%), the effectiveness of amylin was compared across diet groups on pregnancy day G11 ± 3. Overall, amylin’s effectiveness to reduce food intake was similar between diet groups, and consistent with what is observed in non-pregnant rats (Boyle et al., 2011). There was a main effect of amylin treatment on food intake at 30 min (*F*_1_,_15_ = 31.82, *P* < 0.001; Figure 3C), 1 h (*F*_1_,_15_ = 14.76, *P* = 0.002; Figure 3D), and 2 h (*F*_1_,_15_ = 13.29, *P* = 0.02; Figure 3E), but never an effect of diet. By 24 h, all treatment effects were gone (Figure 3F).

#### Maternal Behavior

A pup retrieval test followed by 60 min of home-cage observations of maternal behaviors was conducted on P2 and P7 in dams fed chow or HE. We observed no effect of maternal diet on the latency to retrieve pups to the nest or begin nursing on P2 (Figure 3G) or P7 (Figure 3I). When using the durations of nursing behavior and eating behavior to approximate pup- and dam-related behavior, we again observed no effect of diet on these parameters on P2 (Figure 3H) or P7 (Figure 3J).

### Experiment Two: Influence of High Energy Diet and Polygenic Predisposition on Postpartum Metabolic and Behavioral Outcomes in F3 Dams

#### Maternal Body Weight Gain and Food Intake

**FIGURE 4:**
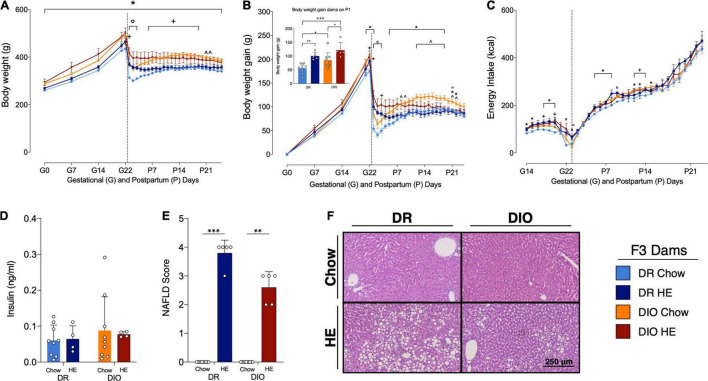
Influence of HE-diet on physiological parameters in diet resistant (DR) and diet induced obese (DIO) rat dams in filial generation 3 (F3) before, during and after pregnancy. Body weight in grams **(A)**, body weight gain in grams **(B)**, and energy intake in kilocalories **(C)** of DR dams on chow (light blue), or HE diet (dark blue), DIO dams on chow (orange), or HE diet (red) during pregnancy and lactation. Vertical dotted line marks parturition. **(B)** Inset shows body weight gain during pregnancy (difference between body weight on G1 and P1. **(D)** Insulin levels (ng/ml) in plasma of DR and DIO dams on chow or HE diet at sacrifice on P21 ± 1. **(E)** Average severity of liver steatosis scores according to the NAFLD scoring system for rodents (Grade 0–4) in DR and DIO dams on chow or HE diet. **(F)** Representative images of H&E stained liver sections. Data are represented as mean ± SD. In **(A–C)**, symbols denote significant differences between specific groups: ° DR-Chow vs. DR-HE; * DR-Chow vs. DIO-Chow; + DR-Chow vs. DIO-HE; ∧ DR-HE vs. DIO-Chow; “DIO-Chow vs. DIO-HE. For all comparisons, the number of symbols signify level of significance: *P < 0.05, **P < 0.01, ***P < 0.001. DR-chow (*N* = 10), DR-HE (*N* = 5), DIO-chow (*N* = 9), and DIO-HE (*N* = 5).

An overall effect of time (*F*_3_._7,91_._4_ = 316.1, *P* < 0.0001), phenotype (*F*_1,25_ = 23.56, *P* < 0.0001), time × phenotype interaction (*F*_29,715_ = 3.549, *P* < 0.0001) and time × diet interaction (*F*_29,715_ = 18.80, *P* < 0.0001) on body weight was found (Figure 4A). DR chow-fed rats had significantly lower body weight compared to DIO chow-fed animals already starting from day G1 until the end of the experiment on P25 (average *P* < 0.001). Furthermore, DR chow-fed animals were significantly lighter than DIO HE-fed animals at P1 (*P* = 0.04). DR HE-fed rats were significantly lighter than DIO chow-fed animals from day P6–P22 (average *P* = 0.01). In addition, DR-chow animals dropped more body weight from P1 to P3 compared to DR HE-fed dams (average *P* = 0.002).

Similar effects were observed for the body weight gain (Figure 4B). There was an overall effect of time (*F*_3_._3,82_ = 332.6, *P* < 0.0001), phenotype (*F*_1,25_ = 12.14, *P* = 0.0018), a time × phenotype interaction (*F*_28,697_ = 3.348, *P* < 0.0001) and a time × diet interaction (*F*_28,697_ = 19.50, *P* < 0.0001). DR chow-fed dams had significantly less body weight gain compared to DIO chow-fed animals from G21-P1 and from P5 to P22 (average *P* = 0.01). DR chow-fed animals showed reduced body weight gain compared to DIO HE-fed animals from G22 to P3 (average *P* = 0.03). From P12 up to P19 DIO chow-fed animals gained significantly more weight compared with DR HE-fed animals (average *P* = 0.02). Comparing body weight gain, chow-fed animals had a greater body weight gain from P1 to P3 in the DR group and from G22 to P3 in the DIO group compared with their HE-fed counterparts, with a stronger effect in the DR strain (DR-Chow vs. DR-HE average *P* < 0.001; DIO-Chow vs. DIO-HE average *P* = 0.03).

Comparison of body weight gain during pregnancy (the difference between body weight on G1 and on P1 to account for litter and placenta weight, See inset in Figure 4B) revealed a phenotype effect (*F*_1,24_ = 8.125, *P* = 0.009) and diet effect (*F*_1,24_ = 21.04, *P* < 0.001), without a significant interaction (*F*_1,24_ = 0.2153, *P* = 0.65). Multiple comparison analysis showed that DR chow-fed animals gained less body weight during pregnancy compared to DR HE-fed dams (*P* = 0.008), as well as compared to DIO chow-fed dams (*P* = 0.05). Further, DIO HE-animals gained more body weigh compared to DIO chow-fed dams (*P* = 0.04). The biggest difference in weight gain could be observed between DR chow-fed dams and DIO HE-fed animals (*P* < 0.001). DIO HE-fed gained 119% more gestational weight during pregnancy compared to the DR chow-fed group.

When food intake was corrected for the energy density per gram food in kilocalories (kcal) there was a time effect (*F*_7_._1,167_._5_ = 431.0, *P* < 0.0001), a diet effect (*F*_1,24_ = 16.98, *P* = 0.0004), a time × diet interaction (*F*_31,727_ = 1.471, *P* = 0.0489) and a phenotype × diet interaction (*F*_1,24_ = 6.380, *P* = 0.0185; Figure 4C). Multiple comparisons detected only few significant differences between groups on specific days. DR chow-fed dams ate significantly less kcal than DR HE-fed animals on day G22 and P9 (average *P* = 0.021). Further, DIO chow-fed rats ate significantly more kcal on G15–G16, G18–G20 and from P5 to P8 compared to DR chow-fed dams, showing a tendency for DIO animals to eat more energy during gestation and lactation (average *P* = 0.015). In addition, HE-fed animals exhibited an attenuated decrease in kcal intake around P1 compared chow-fed to animals (DR-Chow vs. DR-HE on G22 *P* = 0.04; DIO-Chow vs. DIO-HE on P1 *P* = 0.01; DR-HE vs. DIO-Chow on P1 *P* = 0.02). On G18 and G20 and from P12 to P14 DIO HE-fed animals ate significantly more kcal compared to DR chow-fed animals (average *P* = 0.36).

#### Postpartum Insulin Levels

Postpartum insulin levels were measured at day of sacrifice around postpartum day 21 ± 1. There was no significant difference in insulin levels between diet or phenotype groups (Figure 4D). Because a majority of dams were treated with leptin prior to sample collection, plasma leptin levels could not be accurately quantified.

#### Histological Analysis of Postpartum Liver

Consistent with observations in the first experiment, there was mild to severe steatosis in all livers from HE-fed lactating dams (average score DR: 3.8 ± 0.45; DIO: 2.6 ± 0.55), while all chow-fed dams displayed no liver steatosis and received a score of zero. Significant differences between groups could be detected using a Kruskal-Wallis test (*P* < 0.001). Dunn’s multiple comparison revealed significant differences between DR chow vs. DR HE (*P* < 0.001) and DIO chow vs. DIO HE (*P* = 0.006) groups (Figure 4E). Representative H&E-stained livers from each group are shown in Figure 4F.

#### Meal Pattern Analysis

**FIGURE 5:**
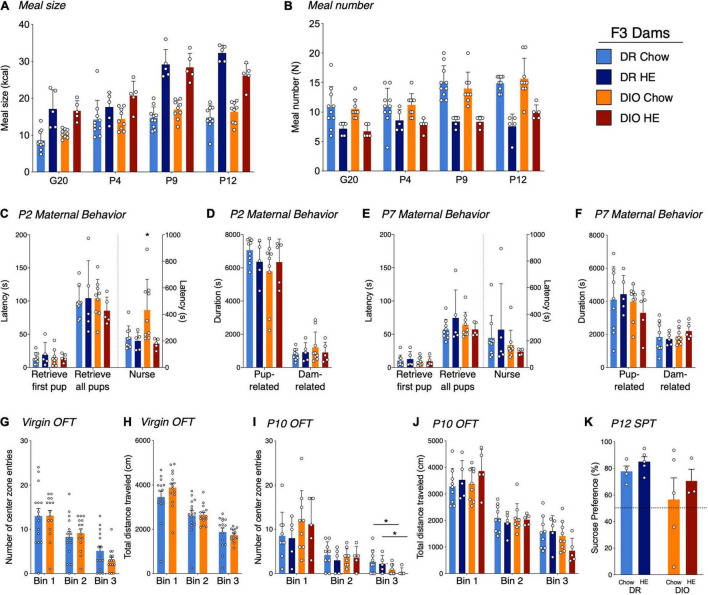
Influence of HE-diet in diet resistant (DR) and diet induced obese (DIO) rat dams in filial generation 3 (F3) on meal pattern, amylin sensitivity and maternal behavior. Average meal size in kilocalories **(A)** and meal number **(B)** of pregnant and postpartum DR and DIO rat dams on chow or high energy (HE) diet on G20, P4, P9, and P12. **(C–F)** Maternal behavior in DR and DIO dams on chow or HE diet on P2 or P7. Latency to retrieve first pup, latency to retrieve all pups and latency to start nursing in seconds on P2 **(C)** and P7 **(E)**. Total duration in seconds of pup-related and dam-related behavior on P2 **(D)** and P7 **(F)**. **(G,H)** Open field test in virgin DR and DIO rat dams around 9 weeks of age on chow. **(G)** Number of center zone entries divided into 3 bins, where one bin represents 5 min. **(H)** Total traveled distance in centimeter (cm) in virgin DR and DIO dams per bin. **(I,J)** OFT on P10 in DR and DIO rat dams on chow or HE diet. Number of center zone entries **(I)** and total traveled distance in centimeter **(J)** divided into 3 5-min bins. **(K)** Sucrose preference in percent of total liquid intake on P14–15 in DR and DIO rat dams on either chow or HE diet. Data are represented as mean ± SD. *P < 0.05. DR-chow (*N* = 10), DR-HE (*N* = 5), DIO-chow (*N* = 9), and DIO-HE (*N* = 5).

Analysis of meal size and meal number revealed the same tendencies as described in generations F0/F1. For meal size (Figure 5A), we detected a main effect of time (*F*_2⋅6, 64⋅6_ = 65.55, *P* < 0.001), diet (*F*_1,25_ = 120.3, *P* < 0.001), time × diet interaction (*F*_3,74_ = 14.68, *P* < 0.001) and time × phenotype × diet interaction (*F*_3,74_ = 3.760, *P* = 0.0143), with animals on HE diet consuming more kcal per meal compared to animals on chow, regardless of phenotype. Meal number (Figure 5B) was significantly lower in HE-fed rats compared to chow-fed rats, again regardless of phenotype. Main effects of time (*F*_2_._680,65_._21_ = 13.86, *P* < 0.001), diet (*F*_1,25_ = 64.88, *P* < 0.001), and time × diet interaction (*F*_3,73_ = 4.894, *P* = 0.0037) were detected.

#### Maternal Behaviors

On P2, while there were no differences between the groups for latency to retrieve pups to the nest, DIO-chow dams demonstrated an increased latency to begin nursing their pups (*P* = 0.0023; Figure 5C), which was significantly higher than all other groups. When total duration of dam- and pup-related behaviors on P2 were compared across groups, no effect of diet or phenotype was observed (Figure 5D). On P7, though there were no differences between latencies to retrieve or nurse (Figure 5E), or the total duration of pup- or dam-related behaviors across groups (Figure 5F), rats on HE diet showed an increased time dedicated to pup grooming compared with chow-fed rats (*F*_1,24_ = 5.281, *P* = 0.03), whereas phenotype had no effect (*F*_1,24_ = 0.02550, *P* = 0.87; See Supplementary Results). On all the other behaviors, and regardless of postpartum day 2 or 7, there were no further statistical differences between the different groups (See Supplementary Results for details). For most behaviors measured, neither phenotype nor diet influenced the maternal behavior on P2 or P7.

#### Open Field Test

To assess the physical activity and anxiety level, rat dams were tested in an open field test prior to pregnancy and again at postpartum day 10 or 11. Latency to enter the center zone, percentage of time spent in the center zone, total distance per 5-min bin, as well as center zone distance and center zone entries per bin were measured. To compare baseline results unpaired *t*-tests were used (Figures 5G,H). There were no statistical differences between DIO and DR animals in any of the assessed parameters. At the time point of baseline measurements, all dams were maintained on chow diet, hence a diet effect could not be measured.

To compare the different parameters on postpartum day 10 or 11, a two-way ANOVA with Tukey’s multiple comparisons test was used (Figures 5I,J). A phenotype effect was observed for the number of center zone entries in Bin 3; DIO rat dams entered the center zone fewer times in the last 5 min of the test, regardless of the diet (*F*_1,24_ = 5.682, *P* = 0.03; Figure 5I). In all other measurements there were no significant differences between phenotype nor diet.

#### Sucrose Preference Test

A sucrose preference test was performed on postpartum day 16. Rats demonstrating a clear side-preference (i.e., consistently drinking from the left or right bottle regardless of content) were excluded from the analysis. There was neither a diet effect (*F*_1,13_ = 0.9778, *P* = 0.34) nor a phenotype effect (*F*_1,13_ = 2.667, *P* = 0.13; Figure 5K) detected.

### Experiment Three: Influence of High Energy Diet and Polygenic Predisposition on Select Postpartum Outcomes in F6 and F7 Dams

#### Maternal Body Weight Gain

**FIGURE 6:**
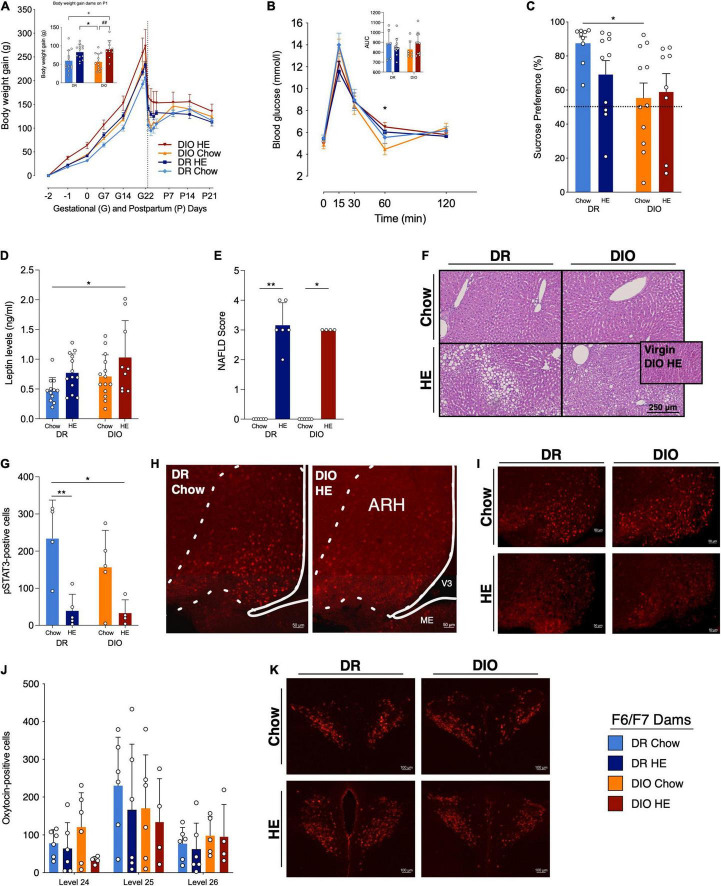
Influence of HE-diet in diet resistant (DR) and diet induced obese (DIO) rat dams in filial generation 6 and 7 (F6/F7) on selected physiological parameters. **(A)** Body weight gain in gram (g) of DR dams on chow (light blue) or HE diet (dark blue), and DIO dams on chow (orange) or HE diet (red) during pregnancy and lactation. Dotted vertical line marks parturition. Inset shows body weight gain during pregnancy (difference between body weight on G1 and P2). **(B)** Blood glucose levels (mmol/l) during an oral glucose tolerance test in DR and DIO dams on chow or HE diet. Inset shows the area under the curve (AUC) relative to zero. **(C)** Sucrose preference in percent of total liquid intake on P14–15 in DR and DIO rat dams on either chow or HE diet. **(D)** Plasma leptin levels in ng/ml in fasted DR and DIO dams on chow or HE diet on P18 ± 1. **(E)** Average severity of liver steatosis according to the NAFLD scoring system for rodents (Grade 0–4) in DR and DIO dams on chow or HE diet. HE diet triggered liver steatosis regardless of phenotype. **(F)** Representative images of H&E sections. Inset in **(F)** shows image from a non-pregnant, non-lactating DIO dam after 6 weeks on HE diet (no steatosis present). **(G)** Quantification of pSTAT3-positive cells in the arcuate nucleus of the hypothalamus (ARH) in DR and DIO dams on chow or HE diet after 5 mg/kg leptin on P21 ± 1. **(H,I)** Representative images of leptin-induced pSTAT3 in the ARH in each group. **(J)** Quantification of oxytocin-positive cells in the paraventricular nucleus of the hypothalamus (PVH) on brain levels 24–26) in DR and DIO dams on chow or HE diet. **(K)** Representative images of the PVH on level 25 in each group. Data are represented as mean ± SD. In the inset in **(A)**, symbols denote significant differences between specific groups: + DR-Chow vs. DIO-HE; * DR-HE vs. DIO-Chow; # DIO-Chow vs. DIO-HE. For all comparisons, the number of symbols signify level of significance: *P < 0.05, **P < 0.01. DR-chow (F6 *N* = 8, F7 *N* = 6), DR-HE (F6 *N* = 7, F7 *N* = 6), DIO-chow (F6 *N* = 8, F7 *N* = 6), and DIO-HE (F6 *N* = 6, F7 *N* = 4).

Select parameters were measured in later generations of the DR and DIO dams. Most data in the following results were collected from F7 dams, however the OGTT, plasma leptin, and SPT also included data from F6 dams. In line with what was observed in the F3 dams, analysis of body weight gain data (Figure 6A) in pregnancy and postpartum revealed main effects of phenotype (*F*_1,15_ = 7.007, *P* = 0.018), diet (*F*_1,15_ = 9.093, *P* = 0.009), and time (*F*_2_._6,38_._2_ = 427.0, *P* < 0.001). Individual group differences detected during late pregnancy were mainly a phenotype effect, meaning that DIO dams gain more weight during pregnancy. The group differences detected in early postpartum were mainly a diet effect, reflecting that chow-fed dams have a more pronounced body weight drop after parturition.

#### Oral Glucose Tolerance Test

Oral glucose tolerance test were conducted in F6 and F7 dams on P18 (Figure 6B). Of note, blood glucose was measured before and after administration of the glucose load via gavage, unlike in F0/F1 dams which consumed the glucose orally. A mixed-effect model analysis of the data revealed a main effect of time (*F*_2_._2,68_._4_ = 142.6, *P* < 0.001), and an interaction between time and diet (*F*_4,124_ = 4.645, *P* = 0.002). While there were no differences in the AUC of the four groups, an individual difference between DIO-Chow and DIO-HE dams was detected at 60 min (*P* = 0.036) with Tukey’s multiple comparisons test.

#### Sucrose Preference Test

While the data from the SPT in F3 dams suggested a trend for DIO dams to exhibit reduced preference for sucrose, a significant main effect of phenotype was observed in the F6/F7 dams (*F*_1,34_ = 6.604, *P* = 0.015), with Tukey’s multiple comparisons test revealing a difference in sucrose preference between the DR-Chow and DIO-Chow dams (*P* = 0.037; Figure 6C). We further calculated the proportion of each group that displayed a sucrose preference, as defined as a sucrose preference ratio < 50%: 100% of the DR-Chow group; 70% of the DR-HE group; 55% of the DIO-Chow group; and 75% of the DIO-HE group.

#### Postpartum Leptin Levels

Following analysis of plasma leptin levels collected at timepoint 0 during the OGTT on P18 (Figure 6D), main effects of phenotype (*F*_1,44_ = 4.891, *P* = 0.032) and diet: (*F*_1,44_ = 7.571, *P* = 0.009) were detected, with Tukey’s multiple comparisons test revealing a significant difference between the leptin levels in DR-Chow vs. DIO-HE dams (*P* = 0.010).

#### Histological Analysis of Postpartum Liver

The presence and extent of steatosis was again assessed in F7 dams, and the results were consistent with the findings from F3 dams. The semi-quantitative scoring (Kruskal-Wallis: *P* < 0.001; Figure 6E) again detected difference between DR-chow and DR-HE (*P* = 0.003) and DIO-chow and DIO-HE (*P* = 0.211) livers. Representative liver images from each group are shown in Figure 6F. Three HE-fed females did not become pregnant but were maintained on HE diet for the duration of the study. Similar to what was observed in the F0/F1 experiment, 100% of livers from the HE-fed non-pregnant females received a score of 0, indicating no hepatic steatosis; one example from this non-pregnant group is presented as an inset in the DIO-HE image in Figure 6E.

#### Postpartum Leptin-Induced pSTAT3 in Arcuate Nucleus of the Hypothalamus

Analysis of the number of leptin-induced pSTAT3-positive neurons in the arcuate nucleus of the hypothalamus (ARH; Figure 6G) in DR and DIO dams fed chow or HE diet, detected a main effect of diet (*F*_1,14_ = 19.13, *P* < 0.001). Multiple comparisons using Tukey’ *post hoc* test, showed that DR-HE dams have less pSTAT3-positive neurons compared to DR-chow (*P* = 0.010), as do DIO-HE compared to DR-chow dams (*P* = 0.011). In Figures 6H,I, representative images of pSTAT3-positive neurons in the ARH Level 29 are shown.

#### Postpartum Levels of Oxytocin in Paraventricular Nucleus of the Hypothalamus

Number of oxytocin-positive cells were evaluated following immunohistochemical staining in the paraventricular nucleus of the hypothalamus (PVH). Figure 6J shows the number of oxytocin-positive neurons in the three brain levels quantified (Level 24–26). Using a three-way ANOVA, we could detect significant differences across different brain levels (*F*_2,53_ = 7.022, *P* = 0.002), however, no further statistical differences could be observed across groups in the PVH at this point of late lactation. In Figure 6K, representative images of oxytocin-positive neurons from PVH Level 25 are shown.

## Discussion

More than one third of women of reproductive age in the United States are obese (Chen et al., 2018). This prevalence of obesity, driven in part by an overconsumption of a palatable, highly caloric diet and a polygenic-sensitivity to such diets, continues to increase (Ward et al., 2019; Wang et al., 2021), further propagating the negative health consequences of maternal obesity for mother and child. Many studies in humans and rodents have investigated the impact of obesity and an unhealthy diet during pregnancy on the offspring. However, fewer studies have evaluated how this metabolic challenge effects the physiological parameters of the mother, despite the evidence that women with obesity are at higher risk for adverse health effects during and after pregnancy, such as gestational diabetes and postpartum depression (Catalano and Ehrenberg, 2006; Molyneaux et al., 2014). The aim of this project was to characterize the physiological and behavioral status of a selectively bred diet-induced obese (DIO) and diet-resistant (DR) rat model fed a high energy diet (HE) or normal chow in a novel context of the metabolically demanding periods of pregnancy and lactation. As our lab was generating new DR and DIO rat lines from outbred Sprague-Dawley rats, we had a unique opportunity to observe the influence of the HE diet and the selective breeding over eight generations of rat dams. Collectively, our studies show that while consumption of HE diet during and after pregnancy can influence maternal physiology and behavior, selective breeding based on the sensitivity to this diet can have both independent and compounded effects on maternal health. Further, this exploratory study provides a foundation for future hypothesis testing using a polygenic model of maternal obesity.

### Effects of Diet and Selective Breeding on Gestational Body Weight and Leptin Levels

A robust diet effect was visible starting in the F0/F1 generation, with HE-fed dams displaying increased gestational body weight and body weight gain compared to chow-fed dams. With further selective breeding of the DIO and DR rat strains, phenotypic differences in gestational body weight and body weight gain were enhanced, as observed in generation F3, with the DIO line having elevated BW and BWG compared to DR dams even in the absence of HE diet. The largest difference in gestational weight gain was observed between DR chow-fed and DIO HE-fed dams in generation F3, with DIO HE-fed animals gaining 119% more weight during pregnancy. Following parturition, dams on chow diet displayed a dramatic drop in body weight, which was followed by a slow but steady regain of body weight mainly from P2 to P12. In contrast, HE dams stayed nearly stable with their body weight after parturition until P25. This effect of HE on the pattern of postpartum body weight changes was observed over several generations, and a similar trend can be observed in recently published work in mouse dams (Moazzam et al., 2021). While the significance of this pattern will require additional probing, the consistency of the effect suggests that intake of HE can influence the metabolic adaptions that occur from pregnancy to postpartum. Consistent with the elevated gestational weight gain, dams fed HE had increased leptin levels during and after pregnancy compared to chow-fed dams. Interestingly, in the later generations of breeding dams (F6/F7), while main effects of diet and phenotype on postpartum leptin levels were observed, there were fewer differences between individual groups. These blunted differences in leptin levels may reflect the reduced impact of phenotype on gestational weight gain in the F6/F7 dams, suggesting that large differences in weight gain potentially peaked in the F3 dams. Based on these observations, we hypothesized that excess adiposity or dietary fat during and after pregnancy, and the resulting increase in maternal levels of circulating leptin, delays or prevents the return of leptin sensitivity in the postpartum period.

### Effects of Diet and Selective Breeding on Leptin Resistance

Leptin resistance is a known and necessary metabolic adaptation that enables maternal fat accumulation during pregnancy (Seeber et al., 2002; Ladyman and Grattan, 2004; Grattan et al., 2007), providing energy to the growing fetus and suckling newborn. The progression of gestational leptin resistance has been documented, and while its reversal after pregnancy is assumed (Andreoli et al., 2019), the time course of its reversal has not been traced. Here we provide initial evidence that intake of HE diet during pregnancy and lactation blunts hypothalamic leptin sensitivity, as demonstrated by reduced leptin-induced pSTAT3 in the brains of HE-fed dams at the time of pup-weaning (P26). We observed this effect of HE in both DR and DIO dams. The precise consequence of retained postpartum leptin resistance has also not been investigated, but could be far-reaching provided the strong connections between metabolic sensing and signaling in the brain and various other brain pathways controlling motivation and cognition (Ferrario et al., 2016; Ge et al., 2018), let alone the impact of leptin signaling in the periphery on energy homeostasis (Vernon et al., 2002). For instance, deficits in postpartum leptin function as a result of HFD-intake in mice was identified as a contributor to insufficient prolactin signaling and lactation (Buonfiglio et al., 2016). And in women, elevated early postpartum leptin levels, which possibly coincide with retained leptin resistance, was shown to be predictive of postpartum depression (Chen et al., 2016).

Leptin resistance during pregnancy and in the postpartum period might underlie other consequences of HE intake in both DR and DIO dams. The meal patterns of HE-fed dams, who consumed significantly larger meals, echoes what we previously observed in leptin receptor-deficient rats (Duffy et al., 2018). Even though the F0/F1 dams largely compensated for the increased caloric density of the HE, showing no differences in 24-h caloric intake except during late pregnancy, a phenotype effect on food intake was detected in the F3 generation. DIO dams on chow consumed slightly more kcal than DR on chow, further confirming our selective breeding methods. An overall diet effect and time × diet interaction was found in dams on HE, who consumed more kcal than animals on chow. These findings are interesting in light of varying postpartum weight change patterns between chow- and HE-fed dams. Despite the relatively similar postpartum caloric intake, dams consuming HE maintained a stable body weight during lactation whereas chow-fed dams exhibited dramatic weight loss followed by regain over the period of lactation. Though we did not evaluate it directly in our studies, one reason for this could involve divergent changes in postpartum energy expenditure. In an earlier study by Wahlig et al. (2012), mice maintained on HFD before and after pregnancy exhibited reduced total body energy expenditure on postpartum day 10, which in combination with increased caloric intake, induced a state of greater positive energy balance in these HFD-fed dams compared to dams fed chow. The authors of this study went on to demonstrate that in dams consuming HFD, dietary fat is used for milk lipid production, rather than energetically costly *de novo* lipogenesis, thus contributing to lower total energy expenditure (Wahlig et al., 2012). Furthermore, as leptin signaling promotes energy expenditure, it is reasonable to hypothesize that leptin resistance, as observed in the HE-fed dams in our study, also plays a role in reduced postpartum energy expenditure.

We further speculate whether a state of postpartum leptin-resistance contributes to the consistent, and often severe, liver steatosis that was observed in dams fed HE diet. Deficient leptin signaling reportedly drives lipid accumulation in the liver, as has been observed in hypoleptinemic people with lipodystrophy (Petersen et al., 2002), and in people with type 2 diabetes and leptin resistance (Cernea et al., 2018). High dietary fat and fructose content are listed as risk factors for liver steatosis in people (Manne et al., 2018), and mice maintained on a 45% HFD for 24 weeks also displayed liver steatosis (Liang et al., 2014). However, our observation that several female rats who failed to become pregnant, but were maintained on the HE for the same duration as the those who did, showed no evidence of hepatic steatosis, suggests that the HE diet interacts with the altered metabolic state of pregnancy or lactation to exacerbate lipid accumulation. Interestingly, a similar phenomenon has been described in high-yielding dairy cows. At lactation onset, some dairy cows can experience extreme energy deficit, which triggers hyper-mobilization of lipids from fat stores, thus causing fatty acid release into the blood stream and their accumulation in the liver (Roberts et al., 1981; Contreras et al., 2010). This extreme state of negative energy balance in cows results from insufficient food intake following parturition, with a concomitant reduction in plasma leptin levels (Block et al., 2001). Consistent with previous work (Vernon et al., 2002; Denis et al., 2003), the chow- and HE-fed rat dams in this study appear to increase their postpartum caloric intake soon after parturition and at a similar rate, yet we postulate that exaggerated or sustained leptin resistance in the HE-fed dams creates a perceived state of negative energy balance, culminating in a hyper-mobilization of fat stores akin to a dairy cow. Chronic infusions of leptin to early lactating cows effectively reduced lipid levels in the liver by 28% (Ehrhardt et al., 2016), lending support to a hypothesis that restoring leptin signaling in HE-fed rat dams would also reduce hepatic steatosis.

### Effects of Diet and Selective Breeding on Glucose Control and Amylin Sensitivity

Overweight and obesity in women is also a known risk factor for gestational diabetes mellitus (Galtier-Dereure et al., 2000). Therefore, we investigated insulin levels and oral glucose tolerance in dams fed chow or HE diet during pregnancy and lactation. While we observed slower glucose clearance in HE-fed F1 dams compared to chow-fed F0 dams, during both pregnancy and lactation, fasting insulin and insulin levels in response to glucose were not statistically different. In the F6/F7 generations, a main effect of phenotype was observed during the postpartum OGTT, with DIO dams on HE demonstrating a delayed glucose clearance compared to DIO-chow dams. To date, most rodent models of gestational diabetes mellitus do not fully recapitulate the human condition – many rely on the use of diabetogenic drugs or manipulation of maternal diet (Holemans et al., 2004; Pasek and Gannon, 2013), and few studies have investigated the impact of gestational diabetes on maternal health beyond the postpartum period (Furse et al., 2021). The data presented here suggest that the current selectively bred DIO model of maternal obesity is not a suitable model of gestational diabetes. However, with further modifications to the maternal diet, such as increasing diet duration or fat or carbohydrate amount or composition, it may present a favorable foundation for future models.

Like leptin, which rises steadily throughout pregnancy, previous studies showed that circulating amylin levels also increase during pregnancy (Kinalski et al., 2004). And while the occurrence of gestational leptin resistance during pregnancy is established (Ladyman et al., 2010), it was not known if this rise in amylin levels also leads to reduced amylin sensitivity, thus providing an additional mechanism to promote increased caloric intake during pregnancy. The treatment of pregnant rat dams with amylin followed by food intake measurements demonstrated that amylin sensitivity is preserved during pregnancy. The effectiveness of amylin to reduce food intake was also not influenced by the diet consumed by the pregnant rats. This finding is consistent with our earlier results, which showed in male rats that obesity or HFD-induced hyperamylinemia did not cause a state of amylin resistance (Boyle et al., 2011).

### Effects of Diet and Selective Breeding on Behavioral Profile of Postpartum Dams

In our first experiments in F0/F1 dams, our lower resolution, real-time analysis of maternal behaviors during a pup-retrieval test following by home-cage observation, failed to detect an effect of HE on maternal behaviors. There was enough variability in these data, however, that left us questioning whether these measures were adequately sensitive to detect potentially subtle changes based on diet. One key aim of the F3 experiment was to perform a high-resolution, blinded analysis of a video-monitored maternal behavior test. Under these experimental conditions, and after three rounds of selective-breeding for obesity-proneness or -resistance, we observed a significant delay in latency to begin nursing in DIO dams. When the test was repeated on P7, when maternal behaviors are consolidated, there were no differences between groups. While these subtle differences are consistent with other published findings in mice that maternal obesity can reduce the quality of maternal care (Bellisario et al., 2015; Bolton et al., 2017; Moazzam et al., 2021), another study in rats showed that HFD increased the time that the dams nursed their pups (Purcell et al., 2011). Because the methods and endpoints used to assess maternal care are not consistent across these or our experiments, it is difficult to determine what are true effects of maternal diet or obesity on maternal care, and what is simply the reflection of natural variations in maternal care that occur over and within the early postpartum days. To bypass such hurdles would require multiple observations periods, if not constant home-cage monitoring, of maternal care. Further, because the home-cage retrieval test is relatively simple for the dams to complete, this again introduces a question of sensitivity. Incorporation of a novel or stress-evoking environment, such as a T-maze retrieval test (Stern and Mackinnon, 1976; Numan, 2020), would further allow us to assess if maternal diet or obesity influences susceptibility or resilience in the face of such a stressor. However, putting these points aside, our blinded analysis of maternal care in F3 dams, suggest that while a polygenic predisposition for obesity might reduce some facets of maternal care, neither intake of HE diet or maternal obesity *per se* reduced the overall quality of maternal care.

Earlier studies reported that rat dams exhibiting high maternal care exhibited higher expression of oxytocin in the PVH, and a higher number of oxytocin receptors in brain centers critical for maternal behaviors (Champagne et al., 2001; Shahrokh et al., 2010). Levels of oxytocin can also be influenced by metabolic state. While leptin was shown to increase PVH oxytocin gene expression (Tung et al., 2008), leptin receptor-deficient rats have reduced plasma oxytocin levels (Gajdosechova et al., 2014). Based on the observed reduction in leptin-sensitivity in the HE-fed dams, we hypothesized that HE-intake would reduce oxytocin expression in the PVH. The quantification of oxytocin-positive neurons in the PVH of DR and DIO rat dams at the time of pup-weaning, however, failed to detect any effect of phenotype or diet on oxytocin levels. During our analysis, we were sensitive to the heterogenous expression pattern oxytocin neurons across the rostro-caudal axis of the PVH (Simmons and Swanson, 2009), only comparing anatomically aligned sections. One important limitation is that we investigated oxytocin immunoreactivity in the PVH, and not the functional release of oxytocin. Interestingly, there is some evidence that leptin can increase activation of oxytocin neurons (Blevins et al., 2004; Perello and Raingo, 2013; Velmurugan et al., 2013), so reduced leptin sensitivity could have an effect on oxytocin function. Another limitation is that we quantified oxytocin at P26, when lactation demands on the dam are falling, in contrast to previous studies that quantified oxytocin on P6 (Shahrokh et al., 2010). So while these data would suggest that metabolic state does not influence late-postpartum levels of oxytocin in PVH, we cannot rule out that metabolic state or leptin sensitivity might influence release of oxytocin into the periphery or at central target sites. Further, we did not investigate whether diet or maternal obesity affect levels of oxytocin receptor expression or sensitivity, in the brain or the periphery. As previous work has demonstrated that oxytocin receptor expression in adipose tissue is impacted by metabolic state (Gajdosechova et al., 2015; Yi et al., 2015), this would be an important direction to pursue.

The lactation period has generally been described as anxiolytic (Neumann, 2001). Rodent dams reportedly spend more time in the open arms of an elevated plus maze (EPM), a readout for reduced anxiety, and this adaptation is thought to be a critical component of the mother’s heightened drive to protect her young. Furthermore, consumption of HFD and weight gain in obesity-prone non-pregnant females was previously shown to increase anxiety-like behavior in EPM (Alonso-Caraballo et al., 2019), leading to the hypothesis that maternal intake of HE would prevent the lactation-driven reduction in anxiety. In our studies, we failed to see robust effects of diet in the open field test. We did not observe phenotype differences in OFT measures in virgin females naïve to HE, nor any global effects of HE or phenotype when the same females were tested on postpartum day 10. The absence of an effect in our study adds to the discrepant findings on the effects of HFD on maternal performance in behavioral paradigms testing anxiety-like behaviors. Moazzam et al. (2021) reported that postpartum mouse dams maintained on HFD spent less time in the closed arms of an EPM than chow-fed dams, which would indicate reduced anxiety. However, a study by Perani et al. (2015) showed that HFD prevented lactation-associated anxiolysis, with HFD-fed mouse dams showing an increase in the latency to enter the lit chamber of the light-dark box, and a decrease in the number of lit chamber entries. Similarly, a study investigated the effects of various maternal diets in mice showed that intake of a diet high in fat and branched-chain amino acids reduced time spent in open arms on P8 (Bolton et al., 2017).

To round out the behavioral profiling of this maternal obesity model, we also performed a mid-lactation sucrose preference test, as a readout for hedonia or pleasure-seeking, in the dams from the F3 and F6/F7 generations. In generation F3, we did not see statistical differences across groups. However, in generations F6/F7, we observed a main effect of both phenotype and diet, with DIO-chow animals showing reduced sucrose preference compared to DR-chow dams. These data are similar to those reported by Moazzam et al. (2021), where pregnant mice on chow displayed higher sucrose preference compared to pregnant mice on HFD. Resistance of the DIO rat dams to consume sweet solution was also observed during the OGTTs performed in the F6 generation, by which we had to abandon the more clinically and physiologically relevant drinking of glucose during the OGTT for gavage administration. The DIO rats, despite an overnight fast prior to the test, simply would not drink the glucose solution. While a reduction in preference for sucrose is a commonly used correlate for an anhedonic or depressed state, it should not be neglected that the test also depends on sucrose detection and post-ingestive learning mechanisms (Sclafani, 2013; Duca et al., 2014). Whether selective breeding based on proneness to weight gain also selects for reduced sucrose preference or detection, in both males and females, would be interesting to investigate. Supporting this, an earlier study demonstrated that obesity-prone male rats maintained on chow demonstrate reduced oral sensitivity to sucrose (Duca et al., 2014).

### Translational Value of the Diet-Resistant/Diet-Induced Obese Model of Maternal Obesity

Herein, we have described the effects of a palatable HE diet in two strains of rats during pregnancy: one that is highly susceptible to weight gain, and one that is resistant. We observed that the intake of HE diet during pregnancy and lactation can have robust effects on various readouts of metabolic health in both strains, and that a polygenic susceptibility to weight gain has an additional impact on some of these measures. Because obesity in women is known to increase the risk of various disorders during and after pregnancy, the polygenic- and diet-sensitive nature of this rodent models provides a multi-faceted tool to dissect how diet, genetics and obesity contribute to these adverse health outcomes. Non-alcoholic fatty liver disease (NAFLD), for example, is more common in women with a BMI above 30 at the start of their pregnancy (Hagstrom et al., 2016). Our data would suggest that maternal diet contributes more toward hepatic steatosis than maternal weight gain or polygenic sensitivity to the diet. The current model would be useful to delineate which components of the HE diet have the largest impact on the liver, and whether HE intake during pregnancy or lactation alone would also produce differential outcomes.

Postpartum depression (PPD) is a devastating and complicated human condition, which also has clear associations with maternal obesity (Kumpulainen et al., 2018; Dachew et al., 2021). Behavioral profiling of the breeding dams suggests that neither maternal obesity nor consumption of a palatable HE led to robust behavioral modifications in the postpartum dam. However, it is interesting to the note that a subset of DIO or HE-fed dams showed delayed behaviors in the maternal tests (i.e., F3 DIO-chow dams on P2) and reduced sucrose preference (i.e., F6/F7 DIO dams, regardless of diet), suggesting modified motivation or reward processing within a susceptible subpopulation of dams. The present rodent model is therefore well-suited to address whether susceptibility for these traits correlate to alterations in neural pathways, such as those involving motivation or stress-responsiveness. This model would also help elucidate what impact certain obesity-related changes (like elevated leptin or leptin resistance) have on the levels of and receptivity to gonadal and metabolic hormones, which are changing so rapidly in the peripartum period.

## Conclusion

In summary, we conclude that intake of HE diet during and after pregnancy can influence maternal physiology and behavior in rat dams and that selective breeding based on the sensitivity to this diet can have both independent and compounded effects on maternal health. Though not without limitations, our unbiased evaluation of maternal behavior showed that HE or propensity to gain weight did not cause major deficits during maternal tests, yet the presence of delayed maternal response and reduced sucrose preference within the DIO and HE-fed groups suggests that these are risk factors for alterations in maternal mood or motivation. This model of maternal obesity, which incorporates both HE diet and polygenic susceptibility for weight gain, represents a useful tool for further probing the biological links between maternal metabolic, reproductive and mental health.

## Data Availability Statement

The raw data supporting the conclusions of this article will be made available by the authors, without undue reservation.

## Ethics Statement

The animal study was reviewed and approved by The Veterinary office of the Canton Zurich, Switzerland.

## Author Contributions

CB contributed to conceptualization. AL, JB, JM, and CB contributed to methodology, investigation, and writing – review and editing. AL, JB, and CB contributed to formal analysis and writing – original draft. All authors contributed to the article and approved the submitted version.

## Conflict of Interest

The authors declare that the research was conducted in the absence of any commercial or financial relationships that could be construed as a potential conflict of interest.

## Publisher’s Note

All claims expressed in this article are solely those of the authors and do not necessarily represent those of their affiliated organizations, or those of the publisher, the editors and the reviewers. Any product that may be evaluated in this article, or claim that may be made by its manufacturer, is not guaranteed or endorsed by the publisher.

## Footnotes

1
https://github.com/DeniseCaiLab/ezTrack
